# Widespread Hepatitis A Outbreaks Associated with Person-to-Person Transmission — United States, 2016–2020

**DOI:** 10.15585/mmwr.mm7139a1

**Published:** 2022-09-30

**Authors:** Monique A. Foster, Megan G. Hofmeister, Shaoman Yin, Martha P. Montgomery, Mark K. Weng, Maribeth Eckert, Noele P. Nelson, Jonathan Mermin, Carolyn Wester, Eyasu H. Teshale, Neil Gupta, Laura A. Cooley, Hepatitis A Response Team, Hepatitis A Response Team, Ryan J. Augustine, Nathan Crawford, D’Angela Green, Yury Khudyakov, Sumathi Ramachandran, Karina Rapposelli, Karena Sapsis, Frank Whitlatch, Melissa A. Morrison, Nakema S. Moss, Priscilla Lauro, Olivia Arizmendi, Jennifer Zipprich, Rachel H. Jervis, Ann Q. Shen, Nikki M. Kupferman, Megan Gumke, Nicole Kikuchi, Ami P. Gandhi, Jared Bartschi, Randi Pedersen, Dawn Nims, Nicole Stone, Lauren Maxwell, Chelsea Raybern, Jennifer Khoury, Amanda Odegård, Raychel Berkheimer, Chloe Manchester, David Blythe, Kompan Ngamsnga, Lindsay Bouton, Erin Mann, Cole Burkholder, Macey Ladisky, Sam Burt, Genny Grilli, Jannifer Anderson, Theresa S. Kittle, Devin Raman, Zuwen Qiu-Shultz, Elizabeth R. Daly, John J. Dreisig, Deepam Thomas, Marla M. Sievers, Jamie Sommer, Cori Tice, Justin Albertson, Susan Sullivan, Brandi Taylor, Lauren Orkis, Kirsten Waller, LaKita Johnson, Rachel Radcliffe, Allison Sierocki, Bree Barbeau, Jeffrey Eason, Kelsey Holloman, Marshall P. Vogt, Mary Chan, Shannon McBee, Melissa Scott

**Affiliations:** ^1^Division of Global Health Protection, Center for Global Health, CDC; ^2^Division of Viral Hepatitis, National Center for HIV, Viral Hepatitis, STD, and TB Prevention, CDC; ^3^Immunization Services Division, National Center for Immunization and Respiratory Diseases, CDC; ^4^National Center for HIV, Viral Hepatitis, STD, and TB Prevention, CDC.; CDC; CDC; CDC; CDC; CDC; CDC; CDC; CDC; CDC; Alabama Department of Public Health; Alabama Department of Public Health; Arizona Department of Health Services; California Department of Public Health; California Department of Public Health; Colorado Department of Public Health and Environment; Colorado Department of Public Health and Environment; Delaware Department of Health and Social Services; Florida Department of Health; Florida Department of Health; Georgia Department of Public Health; Idaho Department of Health and Welfare; Idaho Department of Health and Welfare; Illinois Department of Public Health; Indiana Department of Health; Kansas Department of Health and Environment; Kansas Department of Health and Environment; Kentucky Department for Public Health; Kentucky Department for Public Health; Louisiana Department of Health; Maine Department of Health and Human Services; Maryland Department of Health; Maryland Department of Health; Massachusetts Department of Public Health; Massachusetts Department of Public Health; Michigan Department of Health and Human Services; Michigan Department of Health and Human Services; Minnesota Department of Health; Minnesota Department of Health; Mississippi State Department of Health; Mississippi State Department of Health; Southern Nevada Health District; Southern Nevada Health District; New Hampshire Department of Health and Human Services; New Hampshire Department of Health and Human Services; New Jersey Department of Health; New Mexico Department of Health; New York State Department of Health; New York State Department of Health; North Carolina Department of Health and Human Services; North Carolina Department of Health and Human Services; Ohio Department of Health; Pennsylvania Department of Health; Pennsylvania Department of Health; South Carolina Department of Health and Environmental Control; South Carolina Department of Health and Environmental Control; Tennessee Department of Health; Utah Department of Health and Human Services; Utah Department of Health and Human Services; Virginia Department of Health; Virginia Department of Health; Washington State Department of Health; West Virginia Department of Health and Human Resources; West Virginia Department of Health and Human Resources.

Hepatitis A is a vaccine-preventable disease typically acquired through fecal-oral transmission. Hepatitis A virus (HAV) infection rates in the United States declined approximately 97% during 1995–2015 after the introduction and widespread pediatric use of hepatitis A vaccines (*1*). Since 2016, hepatitis A outbreaks have been reported in 37 states, involving approximately 44,650 cases, 27,250 hospitalizations, and 415 deaths as of September 23, 2022 (*2*). A report describing early outbreaks in four states during 2017 noted that most infections occurred among persons reporting injection or noninjection drug use or experiencing homelessness; this finding signaled a shift in HAV infection epidemiology from point-source outbreaks associated with contaminated food to large community outbreaks associated with person-to-person transmission (*3*). CDC analyzed interim data from 33 outbreak-affected states to characterize demographic, risk factor, and clinical outcome data from 37,553 outbreak-associated hepatitis A cases reported during August 1, 2016–December 31, 2020. Among persons with available risk factor or clinical outcome information, 56% reported drug use, 14% reported experiencing homelessness, and 61% had been hospitalized; 380 outbreak-associated deaths were reported. The most effective means to prevent and control hepatitis A outbreaks is through hepatitis A vaccination, particularly for persons at increased risk for HAV infection (*4*). The epidemiologic shifts identified during these outbreaks led to a 2019 recommendation by the Advisory Committee on Immunization Practices (ACIP) for vaccination of persons experiencing homelessness and reinforcement of existing vaccination recommendations for persons who use drugs (*4*). Substantial progress in the prevention and control of hepatitis A has been made; the number of outbreak-affected states has been reduced from 37 to 13 (*2*). Increased hepatitis A vaccination coverage, particularly through implementation of successful, nontraditional vaccination strategies among disproportionately affected populations (*5*), is needed to continue progress in halting current outbreaks and preventing similar outbreaks in the future.

Health departments investigated HAV infections among persons who met the Council of State and Territorial Epidemiologists’ hepatitis A case definition^†^ using state-specific case investigation forms. Deidentified demographic, risk factor, and clinical outcome data were requested from all states reporting outbreaks for all outbreak-associated cases during August 1, 2016–December 31, 2020. Risk factors were assessed during the exposure period (15–50 days before symptom onset). States were excluded from variable-specific analysis of any variable with 100% missing data. The analysis was conducted using SAS (version 9.4; SAS Institute). Data collection, which was directly related to disease control, was deemed not to be human subjects research. This activity was reviewed by CDC and conducted consistent with applicable federal law and CDC policy.^§^

CDC analyzed data from 33 of 36 (92%) outbreak-affected states^¶^ that were eligible for inclusion** (Figure); these 33 states accounted for approximately 97% of publicly reported hepatitis A outbreak-associated cases at the end of 2020 (*4*). Among 37,553 reported cases, most were among males (62%), White persons (81%), and those aged 30–49 years (58%) (Table). Median age was 38 years. Among cases with data available, 5% and 30% had evidence of past or current hepatitis B or hepatitis C virus infection, respectively; 61% of persons with hepatitis A were hospitalized, and 1% died. Among persons with outbreak-associated HAV infection and available risk factor information, 56% reported injection or noninjection drug use, 14% reported experiencing homelessness, 12% reported recent incarceration, and 3% reported recent international travel; 5% of males self-identified as men who have sex with men.

**FIGURE:**
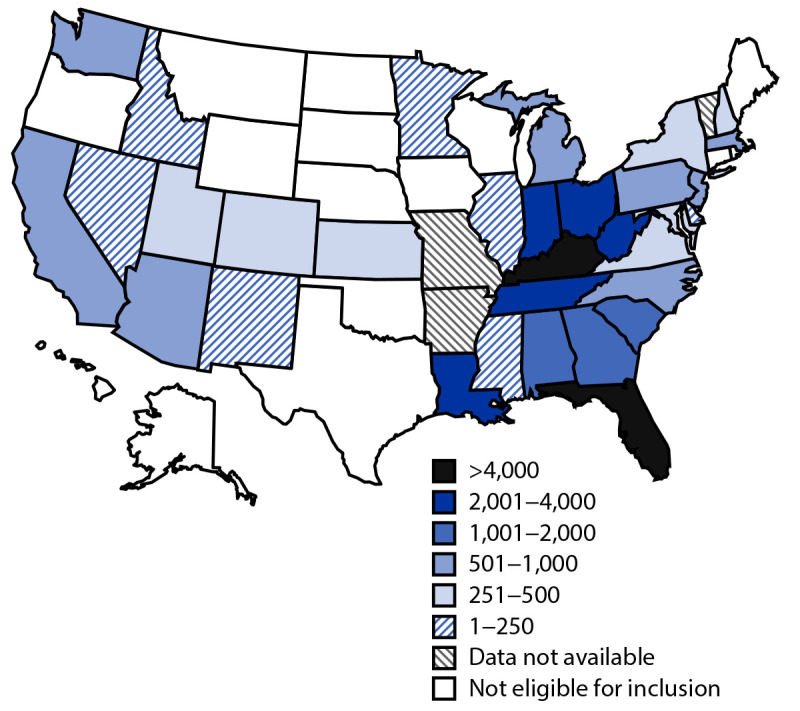
Cumulative outbreak-associated hepatitis A cases reported, by state* — United States, August 1, 2016–December 31, 2020 * States were eligible for inclusion if, as of the initial request for data in August 2020, they had declared a hepatitis A outbreak associated with person-to-person transmission at any point since August 1, 2016.

**TABLE:** Characteristics of outbreak-associated hepatitis A cases — United States, August 1, 2016–December 31, 2020

Characteristic (no. with available information*)	No. (%)
**Total**	**37,553**
**Sex** (37,553)	
Female	14,205 (37.8)
Male	23,317 (62.1)
Other	11 (0)
Missing	20 (0.1)
**Age group, yrs** (37,553)	
0–9	114 (0.3)
10–19	395 (1.1)
20–29	7,130 (19.0)
30–39	13,088 (34.9)
40–49	8,583 (22.9)
50–59	5,082 (13.5)
≥60	3,099 (8.3)
Missing	62 (0.2)
**Race^†^** (21,952)	
American Indian or Alaska Native	103 (0.5)
Asian or Pacific Islander	186 (0.8)
Black or African American	1,438 (6.6)
White	17,831 (81.2)
Other	693 (3.2)
Missing	1,701 (7.7)
**Hospitalized** (37,553)	
Yes	23,043 (61.4)
No	12,770 (34.0)
Missing	1,740 (4.6)
**Death^§^** (37,071)	
Yes	380 (1.0)
No	26,013 (70.2)
Missing	10,678 (28.8)
**Any drug use** (37,553)	
Yes	20,991 (55.9)
No	10,268 (27.3)
Missing	6,294 (16.8)
**Injection drug use^¶^** (22,645)	
Yes	8,601 (38.0)
No	8,250 (36.4)
Missing	5,794 (25.6)
Noninjection drug use** (22,088)	
Yes	7,754 (35.1)
No	7,849 (35.5)
Missing	6,485 (29.4)
**Homelessness^††^** (36,311)	
Yes	5,008 (13.8)
No	15,383 (42.4)
Missing	15,920 (43.8)
**Recent incarceration^§§^** (27,404)	
Yes	3,231 (11.8)
No	14,035 (51.2)
Missing	10,138 (37.0)
**Men who have sex with men^¶¶^** (20,973)	
Yes	1,129 (5.4)
No	7,477 (35.7)
Missing	12,367 (59.0)
International travel*** (26,466)	
Yes	793 (3.0)
No	15,686 (59.3)
Missing	9,987 (37.7)
**Hepatitis B coinfection^†††^** (20,592)	
Yes	1,076 (5.2)
No	7,242 (35.2)
Missing	12,274 (59.6)
**Hepatitis C coinfection^§§§^** (21,357)	
Yes	6,470 (30.3)
No	5,684 (26.6)
Missing	9,203 (43.1)
**Hepatitis B or hepatitis C coinfection^¶¶¶^** (23,937)	
Yes	7,480 (31.2)
No	7,327 (30.6)
Missing	9,130 (38.1)

* States were excluded from variable-specific analysis of any variable with 100% missing data. The number with available information was used as the denominator for percent calculations for each characteristic.

^†^ Twenty-seven states contributed data on race (Alabama, California, Colorado, Delaware, Florida, Georgia, Idaho, Illinois, Kansas, Louisiana, Maine, Maryland, Massachusetts, Minnesota, Mississippi, Nevada, New Hampshire, New Jersey, New Mexico, New York, North Carolina, Pennsylvania, South Carolina, Tennessee, Utah, Virginia, and Washington).

^§^ Thirty-two states contributed data on death (Alabama, Arizona, California, Colorado, Delaware, Florida, Georgia, Idaho, Illinois, Indiana, Kansas, Kentucky, Louisiana, Maine, Maryland, Massachusetts, Michigan, Minnesota, Mississippi, Nevada, New Hampshire, New Jersey, New Mexico, North Carolina, Ohio, Pennsylvania, South Carolina, Tennessee, Utah, Virginia, Washington, and West Virginia).

^¶^ Twenty-six states contributed data on injection drug use (Alabama, Arizona, California, Colorado, Delaware, Florida, Georgia, Idaho, Illinois, Kansas, Louisiana, Maine, Maryland, Massachusetts, Minnesota, Mississippi, Nevada, New Hampshire, New York, North Carolina, Pennsylvania, Tennessee, Utah, Virginia, Washington, and West Virginia).

** Twenty-four states contributed data on noninjection drug use (Alabama, Arizona, California, Colorado, Delaware, Florida, Georgia, Idaho, Illinois, Kansas, Louisiana, Maine, Maryland, Massachusetts, Minnesota, New Hampshire, New York, North Carolina, Pennsylvania, Tennessee, Utah, Virginia, Washington, and West Virginia).

^††^ Thirty states contributed data on homelessness (Alabama, Arizona, California, Colorado, Delaware, Florida, Georgia, Idaho, Illinois, Indiana, Kansas, Kentucky, Louisiana, Maryland, Massachusetts, Michigan, Mississippi, Nevada, New Hampshire, New Jersey, New Mexico, New York, North Carolina, Ohio, South Carolina, Tennessee, Utah, Virginia, Washington, and West Virginia). Homelessness was categorized to include those meeting the U.S. Department of Housing and Urban Development definition of “Literally Homeless” (https://files.hudexchange.info/resources/documents/HomelessDefinition_RecordkeepingRequirementsandCriteria.pdf) as well as those who were unstably housed (e.g., “couch surfing”).

^§§^ Twenty-five states contributed data on recent incarceration (Alabama, Arizona, California, Colorado, Delaware, Florida, Georgia, Idaho, Indiana, Louisiana, Maryland, Massachusetts, Minnesota, Mississippi, Nevada, New Hampshire, New Jersey, New York, North Carolina, Ohio, Pennsylvania, South Carolina, Tennessee, Utah, and Washington).

^¶¶^ Restricted to males; 31 states contributed data on men who have sex with men (Arizona, California, Colorado, Delaware, Florida, Georgia, Idaho, Illinois, Indiana, Kansas, Kentucky, Louisiana, Maine, Maryland, Massachusetts, Michigan, Minnesota, Mississippi, Nevada, New Hampshire, New Jersey, New Mexico, New York, North Carolina, Ohio, Pennsylvania, South Carolina, Tennessee, Utah, Virginia, and Washington).

*** Twenty-four states contributed data on international travel (Arizona, California, Delaware, Florida, Georgia, Idaho, Illinois, Indiana, Kentucky, Maine, Maryland, Massachusetts, Michigan, Mississippi, Nevada, New Hampshire, New Mexico, New York, Pennsylvania, South Carolina, Tennessee, Utah, Virginia, and Washington).

^†††^ Nineteen states contributed data on hepatitis B coinfection (California, Delaware, Georgia, Kansas, Kentucky, Louisiana, Maine, Massachusetts, Michigan, Minnesota, New Hampshire, New Jersey, New Mexico, North Carolina, Ohio, Pennsylvania, Utah, Washington, and West Virginia).

^§§§^ Twenty-one states contributed data on hepatitis C coinfection (California, Colorado, Delaware, Georgia, Kansas, Kentucky, Louisiana, Maine, Maryland, Massachusetts, Michigan, Minnesota, New Hampshire, New Jersey, New Mexico, North Carolina, Ohio, Pennsylvania, Utah, Washington, and West Virginia).

^¶¶¶^ Twenty-two states contributed data on hepatitis B or hepatitis C coinfection (California, Colorado, Delaware, Georgia, Indiana, Kansas, Kentucky, Louisiana, Maine, Maryland, Massachusetts, Michigan, Minnesota, New Hampshire, New Jersey, New Mexico, North Carolina, Ohio, Pennsylvania, Utah, Washington, and West Virginia).

## Discussion

Since 2016, the United States has experienced widespread hepatitis A outbreaks associated with person-to-person transmission. Interim data from 33 states were analyzed to characterize demographic, risk factor, and clinical outcome data from 37,553 outbreak-associated cases reported during August 1, 2016–December 31, 2020. Cases occurred predominantly among males, White persons, and those aged 30–49 years. The most frequently reported risk factor was drug use.

These outbreaks mark a shift in hepatitis A epidemiology in the United States. Before the introduction of hepatitis A vaccines, HAV transmission was driven largely by spread from asymptomatically infected children, and hepatitis A disproportionately affected racial and ethnic minority populations (*6*). In these recent hepatitis A outbreaks associated with person-to-person transmission, however, fewer than 1% of cases occurred among persons aged <18 years, and among cases with available race data, more than 80% occurred among White persons. Whereas international travel and exposure to foodborne outbreaks were previously the most frequently reported risk factors (*7*), drug use (both injection and noninjection) was the predominant risk factor associated with HAV transmission during the 2016–2020 outbreaks. HAV transmission among persons who use drugs occurs through the fecal-oral route (e.g., resulting from lack of sanitation or poor hygiene practices) and might occur percutaneously during injection drug use (*3*).

Sixty-one percent of persons were hospitalized during the hepatitis A outbreaks associated with person-to-person transmission, which substantially exceeds the proportion of hospitalized cases historically reported in the National Notifiable Diseases Surveillance System (NNDSS); in 2016, 42% of persons with hepatitis A cases reported to NNDSS were hospitalized (*8*). The older age of patients and corresponding increased likelihood of comorbidities (including coinfection with hepatitis B or hepatitis C virus in nearly one third of cases) likely contributed to the higher prevalence of hospitalization observed in the recent and ongoing hepatitis A outbreaks. Hospitalization and death from HAV infection occur more frequently among adults than among children (*9*).

The outbreaks described in this report are unprecedented in the hepatitis A vaccine era. National Health and Nutrition Examination Survey data obtained during 2011–2016 indicated that more than 60% of U.S.–born, noninstitutionalized civilian adults in risk groups recommended to receive hepatitis A vaccine by ACIP since 1996 remained susceptible to HAV infection (*10*). Proactive vaccination of adults at increased risk for HAV infection or adverse consequences of infection is critical to prevent outbreaks and serious illness.

In collaboration with state and local health departments, CDC launched a large-scale, multidisciplinary response in 2017 to control the ongoing outbreaks associated with person-to-person transmission. To provide hepatitis A vaccination to disproportionately affected populations most affected by the outbreaks, health departments developed and implemented nontraditional vaccination and staffing strategies (*5*). These included holding satellite vaccination clinics (e.g., at correctional facilities, substance use treatment facilities, syringe services programs, and homeless shelters) and broadening the scope of health care professionals approved to administer vaccines. To overcome barriers to vaccination, including mistrust, stigma, and vaccine hesitancy, health departments partnered with organizations that have long-standing, trusted relationships with persons at risk for HAV infection (*5*). In September 2022, as a result of these intensive and innovative efforts, 24 states have officially declared their outbreaks over, and the remaining 13 states report decreased case counts from the peaks of their outbreaks (*2*).

The findings in this report are subject to at least five limitations. First, risk factor data were self-reported and subject to recall and social desirability biases. Second, hepatitis A surveillance in the United States is passive; thus, case counts might underestimate the actual number of cases. Third, a substantial proportion of data was missing; caution should be exercised when interpreting results with high rates of missing data. Fourth, ethnicity was not systematically ascertained and could not be included. Finally, states did not use an identical hepatitis A–related death case classification, which might have resulted in differential classification of deaths as being hepatitis A–related.

Hepatitis A epidemiology in the United States has shifted as a result of the ongoing outbreaks associated with person-to-person transmission. Cases occurred almost exclusively among adults, and HAV transmission was driven primarily by close contact among persons who use illicit drugs and persons experiencing homelessness. Improving services for these populations, including access to substance use treatment and sanitation, are important considerations in mitigating HAV transmission. Many adults at increased risk for HAV infection remain vulnerable to infection, despite long-standing vaccination recommendations. Given the high hospitalization rate during these outbreaks and the high level of susceptibility to HAV infection among adults in the United States, efforts are needed to improve awareness of and adherence to ACIP hepatitis A vaccination recommendations. Increased hepatitis A vaccination coverage, through implementation of nontraditional vaccination strategies to reach disproportionately affected populations, along with improved universal and catch-up childhood vaccination, will be necessary to respond to the current hepatitis A outbreaks and prevent similar outbreaks in the future. Lessons learned during these outbreaks have been reinforced by experiences during the COVID-19 pandemic and other vaccine-preventable disease outbreaks. Disproportionately affected populations often experience stigma, mistrust, and societal barriers that limit adequate access to the health care system. Continued improvements in vaccination infrastructure, immunization information systems, and education and outreach are critically needed to build vaccine confidence and improve vaccine delivery in nontraditional settings.

**Summary**

**What is already known about this topic?**

Hepatitis A cases declined substantially in the United States after the introduction of hepatitis A vaccines in 1996.

**What is added by this report?**

Hepatitis A epidemiology in the United States has shifted as a result of recent and ongoing outbreaks associated with person-to-person transmission. During August 1, 2016–December 31, 2020, 33 states reported hepatitis A outbreaks involving approximately 37,500 cases. Among cases with available information, 56% of persons reported drug use, 14% reported homelessness, and 61% were hospitalized; 380 outbreak-associated deaths were reported.

**What are the implications for public health practice?**

Increased hepatitis A vaccination coverage, through implementation of nontraditional vaccination strategies to reach disproportionately affected populations, along with improved universal and catch-up childhood vaccination, will be necessary to respond to the current hepatitis A outbreaks and prevent similar outbreaks in the future.

## Footnotes

* These authors contributed equally to this report.

†
https://ndc.services.cdc.gov/conditions/hepatitis-a-acute/

§ 45 C.F.R. part 46.104.

¶ The 33 outbreak-affected states included in the analysis were Alabama, Arizona, California, Colorado, Delaware, Florida, Georgia, Idaho, Illinois, Indiana, Kansas, Kentucky, Louisiana, Maine, Maryland, Massachusetts, Michigan, Minnesota, Mississippi, Nevada, New Hampshire, New Jersey, New Mexico, New York, North Carolina, Ohio, Pennsylvania, South Carolina, Tennessee, Utah, Virginia, Washington, and West Virginia.

** States were eligible for inclusion if, as of the initial request for data in August 2020, they had declared a hepatitis A outbreak associated with person-to-person transmission at any point since August 1, 2016.
